# Development and preliminary validation of the nutrition literacy scale for type 2 diabetes mellitus in China—a mixed methods study

**DOI:** 10.3389/fpubh.2025.1569675

**Published:** 2025-05-13

**Authors:** Wenjuan Zhang, Yan Wu, Yibao Zhang, Ziyu Sun, Jiaqi Wang, Yuhong Wu

**Affiliations:** ^1^School of Public Health and Nursing, Hangzhou Normal University, Hangzhou, China; ^2^Department of Endocrinology, Zhejiang Greentown Cardiovascular Disease Hospital, Hangzhou, China

**Keywords:** diabetes mellitus, literacy, nutrition status, scale, validation

## Abstract

**Background:**

Nutrition literacy is an important predictor of eating behavior in patients with type 2 diabetes. However, current methods lack rigorously validated tools to assess nutrient literacy in this group. Therefore, the aim of this study was to develop and validate the Nutrition Literacy Scale for patients with type 2 diabetes mellitus.

**Methods:**

This study was divided into two phases, including the generation of the items and the validation of the scales. The generation of the items was developed through a literature study, semi-structured interviews, a Delphi expert consultation, and a small-sample pre-survey. Convenience sampling method was used to select a large sample of 576 patients in Hangzhou for item analysis, reliability and validity tests.

**Results:**

The formal scale covered 4 dimensions with 31 items. The exploratory factor analysis extracted four common factors with a cumulative variance contribution of 62.725%; the results of the confirmatory factor analysis showed that the model fit was good, the content validity of the scale was 0.957. The calibration correlation validity of the newly developed scale with the Adult Nutrition Literacy Measurement Scale was 0.760. Cronbach’s *α* coefficient was 0.946, and re-test reliabilitiy was 0.884.

**Conclusion:**

The nutrition literacy scale for people with type 2 diabetes has good reliability and validity and is suitable for assessing the level of nutrition literacy in relevant populations.

## 1 Introduction

Type 2 diabetes is a long-term metabolic syndrome characterized by hyperglycaemia, insulin resistance and relative insulin deficiency. According to the 10th edition of the International Diabetes Federation (IDF) Diabetes Map (1), as of 2021, the number of diabetes patients worldwide has reached 537 million, of which 140 million are already in China, ranking first in the world, with type 2 diabetes accounting for about 90% of all types of diabetes (2).

Nutrition therapy and dietary management, the cornerstone of the type 2 diabetes care system, is present throughout the management of the disease and is a key component in maintaining stable blood glucose levels and slowing the progression of complications (3). However, the reality is not optimistic, as patients with type 2 diabetes generally show low adherence in following nutrition management instructions and improving self-management (4), which not only affects the treatment outcome, but also increases the difficulty of disease management. Nutrition literacy is the ability of an individual to acquire, process, understand and apply nutrition information and skills, and to make appropriate nutrition decisions (5). Nutrition literacy has been shown in several studies to be a predictor of healthy dietary choices in people with chronic diseases, and can help them improve diet quality and adherence (6–10). Therefore, assessing the level of nutrition literacy in patients with type 2 diabetes is crucial to improve patients’ disease outcomes.

## 2 Background

Currently, the most widely used tool in the field of chronic diseases is the Nutrition Literacy Assessment Tool (NLAT) (10, 11), which has been translated and culturally adapted by scientists from Spain, Italy, China and other countries, and has been validated and used in populations with diabetes, hyperlipidaemia, hypertension and overweight or obese people. However, the scale dimensions are limited to the functional level and do not yet take into account patients’ willingness to receive diet and nutrition information and the interactive critical level. Besides, the scale items do not take into account the diet and nutrition-related content of diabetic patients, which lacks the specificity of nutrition literacy assessment in type 2 diabetic patients.

Other specific scales, including the nutrition Literacy Measurement Tool for End-stage Renal Dialysis Patients (NLMTERDP) (12) and the Nutrition Literacy Scale for Peritoneal Dialysis Patients (NLSPDP) (13) have been validated and applied in China, which is more targeted, has good reliability and validity in the target population, and is able to accurately respond to the level of nutrition literacy in the measured population.

In conclusion, existing assessment tools are not specific for a comprehensive reflection of the level of nutrition literacy in patients with type 2 diabetes. Therefore, the aim of this study was to develop and test the reliability of a comprehensive nutrition literacy scale for patients with type 2 diabetes. This can help nurses develop more targeted interventions and evaluate the effect of interventions.

## 3 Methods

### 3.1 Research design

The Consensus-Based Standards for the Selection of Health Measurement Instruments (COSMIN) was employed (14). This study is divided into two phases: ① Develop the pretest version of the scale ② Improve and psychometric evaluation of the scale (15).

### 3.2 Theoretical framework

In this study, the Health Literacy Stratification Model (16) and the Knowledge, Belief and Action Theory (17) were combined to develop the nutrition literacy scale for patients with type 2 diabetes. The combination of the two theories is specified in that functional nutrition literacy focuses on knowledge, which tends to be the ‘knowledge’ of the Knowledge-Believe-Action Theory, whereas interactive and critical nutrition literacy focuses on behavior, which tends to be the ‘Action’ of the Knowledge-Believe-Action Theory. The missing aspect of beliefs in the Health Literacy Stratification Model is addressed by the Knowledge-Believe-Action Theory.

### 3.3 Procedure

#### 3.3.1 Phase 1: development of the pretest version of the scale

##### 3.3.1.1 Literature review

Pub Med, Web of Science, Embase, CINAHL, Scopus, Cochrane, JBI, CNKI, Wanfang databases were searched. The search term is “type 2 diabetes mellitus, diabetes mellitus, diabetes,” “nutrition, nutrition literacy, health literacy.” We used the retrieval method of free words and subject words, and the retrieval time was for the self-built library until March, 2024. After reading, the two researchers jointly extracted the information related to sarcopenia, and summarized the common and representative indicators.

##### 3.3.1.2 Semi-structured interview

We used convenience sampling to select patients with type 2 diabetes in a tertiary hospital in Zhejiang Province as interview participants in March–April 2024. Inclusion criteria: ① meets the diagnostic criteria for type 2 diabetes (18); ② age≥18 years old; ③ have a certain level of verbal communication ability; ④ have no obvious cognitive dysfunction; ⑤ signed the informed consent form and have a certain degree of motivation for this study, and be able to complete the survey. Exclusion criteria: type 2 diabetes patients with combination of other critical diseases and unstable condition.

The interview outline is as follows: ① did you have any changes in your diet before and after the illness? Can you give some examples? ② what difficulties and problems do you have with your daily diet and nutrition management? ③ what nutrition information do you pay more attention to in your daily life for better disease management? ④ what skills or qualities do you think are needed in the process of dietary management?

Prior to the interview, the researcher introduced the patient to the purpose and significance of the study, informed the patient that the interview would be audio-recorded in its entirety, and obtained the patient’s consent. The duration of the interview was 15–30 min for each patient. Based on the principle of data saturation in qualitative research as a criterion for sample size, interviews should be stopped when no new themes emerge (19). The interviewers transcribed the audio recordings into text within 24 h of the interviews and analysed the data using Colaizzi’s method.

##### 3.3.1.3 Delphi expert inquiry

We invited 12 experts from different organizations from May to June 2024 for consultation. Inclusion criteria for experts: ① ≥ 10 years of work experience in diabetes mellitus-related specialties in tertiary hospitals/universities; ② master’s degree or higher; ③ associate’s degree or higher; ④ informed consent and willingness to participate in this study. Withdrawal criteria: ① those unable to return revisions on time for the duration of the study; ② those unable to continue for various reasons. An average importance score≥3.5 and a coefficient of variation≤0.25 were used as inclusion criteria (19). We combined expert opinions to form the first draft of scale.

##### 3.3.1.4 Pre-survey

To test whether the presentation of the scale was sufficiently clear to read and answer. A pre-survey was conducted in 24 patients with type 2 diabetes. We investigated the understanding of the content of the items and the difficulty of filling in the items, and recorded the problems and suggestions existing in the process of filling in finally, the final pretest version of the scale was formed.

#### 3.3.2 Phase 2: refinement and psychometric evaluation of the scale

##### 3.3.2.1 Participant and sample size

Between July and October 2024, patients with type 2 diabetes from two tertiary general hospitals in Hangzhou, Zhejiang province, were selected as participants using convenience sampling. The inclusion criteria were the same as those used for the semi-structured interviews. Sample size estimation: Exploratory factor analysis (EFA) was used to test the validity of our scale. The required sample size is 5–10 times the number of items (20). At the same time, the confirmatory factor analysis (CFA) calculates a sample size of >200. The pretest version of the scale consists of 37 items. Taking into account 20% of invalid questionnaires, the sample size must be estimated at 231–462 patients. This study received approval from a local university’s Institutional Review Board (IRB number: 2024062), and all subjects signed informed consent forms.

##### 3.3.2.2 Questionnaires

① General information questionnaire: including age, gender, marital status, education level, monthly income, and mode of residence, etc. ② Nutrition Literacy Scale for Patients with Type 2 Diabetes (pretest version): the questionnaire used Likert 5 level score method," Very disagree/rarely,” “disagree/occasionally,” “dimness/generally,” “agree/often,” “very agree/always” score of 1, 2, 3, 4, 5. Higher scale scores indicate higher nutrition literacy in patients with type 2 diabetes.

##### 3.3.2.3 Data collection

Before the investigation, the researcher will train the research team members to fill in the questionnaire, adopt the unified instruction to inform the purpose and significance of the study, and put the questionnaire to fill it in by themselves. For those with difficulty due to low vision and other reasons, the researcher will ask the offspring one by one.

##### 3.3.2.4 Data analysis

Data analysis was performed using SPSS26.0 and AMOS24.0 software. Continuous variables that adhered to a normal distribution were presented as mean ± standard deviation (SD) and use frequency and percentage for categorical variables.

###### 3.3.2.4.1 Item analysis

① the critical ratio method was used to evaluate the discriminatory nature of the items, and the total scores of the items were ranked from the highest to the lowest, obtaining the scores of the two groups of study subjects in the top 27.00% and the bottom 27.00% of the scores, comparing the scores of the items of the two groups, and retaining those with a decision value of ≥3.00 and *p* < 0.05 (21). ② the correlation between the score of each item and the total scale score was evaluated using the correlation coefficient method, retaining the items with a correlation coefficient >0.40 and *p* < 0.05 (21). ③ the internal consistency coefficient method was used to test internal consistency, and if the Cronbach’s *α* coefficient of the total table became significantly larger after deletion of an item, that item was deleted (22).

###### 3.3.2.4.2 Content validity

Delphi survey used to assess the content of the scale validity. Using Likert 4 grade score, 1 is irrelevant,2 is weak related,3 is strong correlation,4 is very related. Item-content validity index (I-CVI) was calculated as the number of experts given a score of 3 or 4 divided by the total number of experts participating in the evaluation. Scale-content validity index (S-CVI) is the mean of values for all items I-CVI of the scale. I-CVI and S-CVI > 0.78 and 0.90, respectively, indicating good content validity (23).

###### 3.3.2.4.3 Construct validity

1. Exploratory factor analysis

EFA and maximum variance orthogonal rotation (The variances of the factors have been maximized to facilitate the interpretation of the factors.) were used to retain entries with≥3 items and factor loadings≥0.40 under the common factor according to the principles of cumulative variance contribution >60% and eigenvalue >1 (24). Multiple loadings of the items with similar loading values were deleted (loadings were all >0.40, and the difference was <0.20) (25).

1. Confirmatory factor analysis

We also performed CFA to verify the fit of the factor structure derived from EFA. Validation factor analysis model fitting criteria: chi-square degree of freedom ratio (χ^2^/df) < 3.00, approximate error (RMSEA) < 0.08, normalized fit index (NFI), unnormalized fit index (TLI), added adaptation index (IFI), comparative fit index (CFI) > 0.90, goodness of fit index (GFI) and adjusted fit index (AGFI) > 0.80 (26).

###### 3.3.2.4.4 Converge validity

Converge validity was evaluated by factor loadings, combined reliability (CR), and average variance extracted (AVE), and factor loadings greater than 0.50; AVE higher than 0.50, and CR higher than 0.60 were also considered acceptable (27).

###### 3.3.2.4.5 Discrimination validity

Discrimination validity was tested by comparing the AVE square root of each factor with the correlation coefficients between that factor and the other factors; if the AVE square root value was greater than the correlation coefficients, the scale was considered to have good discriminant validity (27).

###### 3.3.2.4.6 Calibration correlation validity

Calibration correlation validity is a test of the correlation between a new scale and the results measured by a standard scale, using a recognized valid scale as the standard. A test with high criterion correlation validity can more accurately reveal the true level or traits of the examinee. In this study, the Nutrition Literacy Measurement Scale for Adults developed by Zhang et al. (28) was used as a validity correlation index, and the two scales were analysed for correlation. *r* was acceptable at 0.4 to 0.8 (13). The scale has been investigated among the adult population in Anhui, China, with a Cronbach’s *α* coefficient of 0.971, split-half reliability of 0.855, and content validity of 0.982. The scale is scored on a 5-point Likert scale, which is more consistent with the themes and scoring method of this study. However, the scale is not disease-specific.

###### 3.3.2.4.7 Reliability

The reliability of the scale is expressed using Cronbach’s *α* coefficient and test–retest reliability. Cronbach’s α coefficient over 0.70 and test–retest reliability over 0.80 indicate acceptable (29).

## 4 Results

### 4.1 Phase 1: development of the pretest version of the scale

#### 4.1.1 Literature review

In the literature review, a total of 4,232 relevant articles were retrieved. After removing duplicates and reviewing the titles, abstracts and full texts in turn, 21 articles were included for detailed analysis. We reviewed and discussed the rationale and wording of each item in the context of clinical practice and in brainstorming sessions with the research team. The first draft of scale was constructed, including 4 dimensions with 34 items.

#### 4.1.2 Semi-structured interview

To ensure that the nutrition literacy scale for people with type 2 diabetes was developed to be more relevant to the clinical setting, structured interviews were conducted to supplement the item pool. With the addition of seven items, these findings have enriched our study. The resulting scale consists of 41 items in 4 dimensions: nutrition belief (6 items), functional nutrition literacy (21 items), interactive nutrition literacy (6 items), and critical nutrition literacy (8 items).

#### 4.1.3 Delphi expert inquiry

In the Delphi expert correspondence, 12 experts participated in the study, including eight master’s degree holders and four PhD holders. In terms of titles, there are four senior titles and eight deputy senior titles. In terms of professional background, they included five diabetic-specialized clinical nurses, two clinicians, three nurse educators, and two chronic disease management specialists. Their average age was 46.41 ± 7.67 years and their average years of work was 18.25 ± 3.67 years.

Finally, the research team organized and discussed the experts’ comments. After the first round of expert consultation. Delete the 4 items. The presentation of 18 items was modified. In the second round of expert consultation, the importance scores of the items ranged from 3.57 to 5.00, with coefficients of variation ranging from 0.090 to 0.124, and there was a convergence of views among the experts. After two rounds of expert correspondence, 3 dimensions and 37 items were retained.

### 4.2 Refinement and psychometric evaluation of the scale

#### 4.2.1 Participants

There were 576 people in total. Table 1 shows the participants’ demographic data, among them 219(38.0%) were female and 357(62.0%) were male. Age was 37.79 ± 13.65 years.

**Table 1:** Participants’ demographic data.

Characteristic	*M* (SD)/*N* (%)
Age	37.79 ± 13.65
Height (CM)	169.56 ± 7.89
Weight (KG)	63.71 ± 11.36
Gender	
Male	357 (62.0%)
Female	219 (38.0%)
Way of living	
Live alone	166 (28.8%)
Living with spouse	269 (46.7%)
Living with children	20 (3.5%)
Living with spouse and children	121 (21%)
Marital status	
Unmarried	177 (30.7%)
Married	384 (66.7%)
Divorced	10 (1.7%)
Widowed	5 (0.9%)
Residence	
Rural	124 (21.5%)
Town	314 (54.5%)
City	138 (24%)
Education level	
Primary school and below	32 (5.6%)
Junior high school	109 (18.9%)
High school or junior college	168 (29.2%)
Colleges and above	267 (46.4%)
Monthly household income (RMB)	
<2000	23 (4.0%)
2000–5,000	164 (28.5%)
>5,000	389 (67.5%)

#### 4.2.2 Content validity

The results show that S-CVI of the scale is 0.957, and I-CVI of each item is 0.833 ~ 1.000, which shows that the content validity of the scale is good.

#### 4.2.3 Item analysis

① Critical ratio method: The decision value for the items ranged from 4.418. ~ 20.861, *p* < 0.05, meeting the retention criteria. ② Correlation coefficient method: Item B1“I know that total energy intake should be controlled,” item B2 “I know that total daily energy can be allocated to the three meals roughly proportionally (1/5 in the morning, 2/5 in the middle of the day, 2/5 in the evening, or 1/3 in each of the three meals),” and item B11 “I know that cooking oil should be limited to 25 g per day” have correlation coefficients of 0.180, 0.291, and 0.262 with the total score of the questionnaire. So consideration should be given to deleting these items. The correlation coefficients between the remaining item scores and the total questionnaire scores ranged from 0.445 to 0.779 (*p* < 0.001). ③ Internal consistency coefficient method: The Cronbach’s *α* coefficient for this stage of the scale was 0.954, after deleting items B1, B2, and B11, the Cronbach’s α coefficient of the questionnaire increased. None of the remaining items exceeded 0.954 and were retained. After item analysis, three items were deleted.

#### 4.2.4 Construct validity

##### 4.2.4.1 Exploratory factor analysis

The remaining 34 items were subjected to the first exploratory factor analysis and the results showed that item B12 “I know I should stop smoking and limit alcohol consumption,” item B21 “I know I should continue to exercise to maintain a healthy weight” and item D2 “I think about whether my diet and exercise are in balance” were multi-factor loadings and the absolute value of the difference in loadings was <0.2. After discussion in the research group, these items were deleted. The remaining 31 items were subjected to a second exploratory factor analysis, which showed that KMO = 0.946, suitable for factor analysis, Bartlett’s test of sphericity χ^2^ = 4815.507 (*p* < 0.001). There were four common factor eigenvalues greater than 1 (13.294, 2.690, 1.993, and 1.467), with a cumulative variance contribution of 62.725%. The factor loading values for each item were >0.50 and there were no cross-loadings, as detailed in Table 2.

**Table 2:** Results of the second exploratory factor analysis.

Items	Factors			
	1	2	3	4
B3 I know that fibre, found in foods such as wholemeal cereals, mixed legumes and vegetables, helps to improve diabetes.	0.753			
B4 I know that whole grains, such as brown rice and oats, should be increased in the diet and refined grains (such as refined white rice and pasta) should be reduced.	0.669			
B5 I know that while controlling total energy, it is appropriate to choose more non-starchy vegetables.	0.710			
B6 I know that while controlling total energy, it is appropriate to choose more fruits with a low glycaemic index.	0.688			
B7 I know that the intake of sucrose and fructose products (e.g., sugar-sweetened drinks) should be strictly controlled.	0.650			
B8 I know that if I want to eat sweets, I can use sugar alcohols or non-nutritive sweeteners (e.g., aspartame, stevioside).	0.822			
B9 I know that I should consume high quality proteins such as eggs, milk, meat and fish as a source of protein daily.	0.646			
B10 I know that fat intake should be controlled (e.g., fatty meats, fried foods, etc.)	0.710			
B13 I know that I can eat nuts that are rich in unsaturated fats (e.g., walnuts, peanuts, etc.).	0.667			
B14 I know that the salt intake should not exceed 5 g a day	0.703			
B15 I know that I should limit my intake of foods high in salt, such as salted vegetables, salted meats and cured meats.	0.730			
B16 I know that similar foods can be interchangeable (such as: pork can be exchanged with chicken, beef, etc.)	0.746			
B17 I know that hypoglycaemia can occur when eating too little/not eating on time	0.735			
B18 I know that I should carry carbohydrate foods (e.g., sugar cubes, biscuits, etc.) with me when I go out and consume them as soon as hypoglycaemia occurs	0.758			
B19 I know that I should drink an adequate amount of water every day (if I have diabetic nephropathy, I know that I should control the amount of water I drink every day)	0.727			
B20 I know it should be cooked more by steaming and boiling	0.632			
B22 I know that I should pay attention to the nutrition labels on the outer packaging when shopping for and eating foods	0.665			
C1 I discuss diabetes nutrition with my family, friends and patients		0.833		
C2 I attended a seminar on diabetes nutrition presented by doctors, nurses, dietitians, etc.		0.838		
C3 I can understand what the professionals say about diabetes nutrition		0.813		
C4 When I have a nutritional problem, I take the initiative to consult a professional		0.733		
C5 I can clearly articulate my diabetic nutritional concerns and recommendations		0.768		
D1 I think about whether my daily diet is scientific and reasonable			0.720	
D3 I think about whether the nutrition information of diabetes in newspapers, magazines and the Internet is scientific			0.622	
D4 I think about whether the diabetes nutrition information provided by my family, friends and patients is scientific			0.675	
D5 I think about whether the diabetes nutrition information provided by food sellers is scientific			0.771	
D6I will choose the right food through my nutrition information and my eating habits and preferences			0.759	
A1 I think diabetes nutrition treatment is very important				0.692
A2 I think nutrition education is necessary for people with diabetes				0.755
A3 I am willing to take the initiative to learn diabetes nutrition knowledge				0.695
A4 I am willing to follow a diabetic diet with or without complications				0.765

##### 4.2.4.2 Confirmatory factor analysis

The results are shown χ2/df = 1.657, RMSEA = 0.044, GFI = 0.953, CFI = 0.953, IFI = 0.954, TLI = 0.949, NFI = 0.890, the model was well fit.

#### 4.2.5 Convergent and discriminant validity

The standardized factor loadings for each item ranged from 0.83 to 0.93. The AVE for nutrition belief was 0.534, functional nutrition literacy was 0.534, interactive nutrition literacy was 0.596, and critical nutrition literacy was 0.576. The CRs for the 4 dimensions were 0.821, 0.951, 0.880, and 0.870, respectively, which gave the scale good convergent validity. The arithmetic square root of the AVE for each latent variable ranged from 0.731 to 0.772, as shown in Table 3, which gave the scale good discriminant validity.

**Table 3:** Discriminant validity.

Variable	Critical nutrition literacy	Interactive nutrition literacy	Functional nutrition literacy	Nutrition belief
Critical nutrition literacy	**0.759**			
Interactive nutrition literacy	0.449***	**0.772**		
Functional nutrition literacy	0.512***	0.455***	**0.731**	
Nutrition belief	0.471***	0.475***	0.635***	**0.731**

^**^* p < 0.001; The bold part is the AVE arithmetic square root of each factor.

#### 4.2.6 Calibration correlation validity

The correlation coefficients between the total score, scores of the dimensions of the nutrition literacy scale for type 2 diabetes and the total score of the Nutrition Literacy Measurement Scale for Adults were 0.760, 0.536, 0.518, 0.566, and 0.674, respectively.

#### 4.2.7 Reliability analysis

The Cronbach’s *α* coefficient for the total scale was 0.946. The Cronbach’s α coefficients for nutrition belief, functional nutrition literacy, interactive nutrition literacy and critical nutrition literacy were 0.819, 0.950, 0.876 and 0.867. The retest reliability of the total scale was 0.884 and the retest reliabilities of the four dimensions were 0.877, 0.928, 0.873 and 0.827.

## 5 Discussion

This study used a standardized and rigorous questionnaire development process to develop nutrition literacy scale for patients with type 2 diabetes. During the development of the questionnaire, the scale was tailored to the actual situation and psychological state of patients, allowing for a more objective assessment of their current level of nutrition literacy for patients with type 2 diabetes. To ensure the rigour and scientific nature of the scale, 12 authoritative experts from different departments and related fields participated in two rounds of Delphi consultation. Our findings confirm that the scale shows good internal consistency and validity. The scale allows healthcare professionals to target interventions to patients with type 2 diabetes with low nutrition literacy scores.

The items “I know that total energy intake should be controlled,” “I know that total daily energy can be allocated to the three meals roughly proportionally (1/5 in the morning, 2/5 in the middle of the day, 2/5 in the evening, or 1/3 in each of the three meals),” and “I know that cooking oil should be limited to 25 g per day” were deleted in the item analysis. This may be due to the fact that most patients were familiar with the emphasis on energy intake control given by health professionals during health education, making it impossible to identify the degree of reflection in different patients. In addition, most patients had some understanding of this content, but exactly how to distribute and intake calculations were more difficult for patients (30).

Bartlett′s spherical test χ^2^ = 4815.507 (*p* < 0.001) and the KMO = 0.946, which was suitable for exploratory factor analysis. Four common factors with eigenvalues >1 were extracted, and the cumulative variance contribution rate was 62.725%, which was much higher than Kidney Scale (12) and Dialysis Scale (13) nutrition literacy scales. In the exploratory factor analyses items “I know I should stop smoking and limit alcohol,” “I know I should continue to exercise to maintain a healthy weight” and “I think about whether my diet and exercise are balanced” were removed. This may be due to the general nature of the above items. There may be differences in understanding and response to the same indicator in different populations, resulting in cross-loading of the same indicator (31). The loadings of remaining items in the corresponding factor were >0.50, and the rotated factor loading matrices were basically consistent with the theoretical framework of scale development, indicating that the scale structure was reasonable. In addition, this scale passed validated factor analyses, content validity, convergent and discriminant validity, calibration correlation validity and reliability tests, which further confirmed that this scale has good reliability and validity.

The original chronic disease pervasive scale (10, 11) only includes a functional nutrition literacy component that focuses on assessing patients’ nutrition knowledge. In this study, we constructed a nutrition literacy scale based on the Health Literacy Stratification Model and the Knowledge, Belief and Action Theory, and combined with the characteristics of patients in their daily dietary management, in order to expand the measurement range of nutrition literacy, with a higher degree of specificity, and focusing on type 2 diabetes patients. The final scale contains 4 dimensions and 31 items.

The scale developed in this study and the Nutritional Literacy Scale for Kidney (12) and Dialysis Patients (13) both included four dimensions of nutritional attitudes, nutritional knowledge reliability, and interaction and criticism of nutritional information, and the Cronbach coefficients of the scales were all >0.8, with good calibration correlations with the relevant standard indicators. However, the Nutritional Literacy Scale for Kidney Patients (12) was not subjected to validated factor analysis, and the structural robustness of the scale still needs to be further tested. In addition, these two scales are population-specific and cannot be used for Chinese patients with type 2 diabetes, a gap that was filled in this study using a standardized process.

Nutrition belief are attitudes that promote the adoption of nutrition health behaviors by individuals guided by their sense of autonomy; functional nutrition literacy refers primarily to knowledge and skills about diabetes nutrition; and interactive critical nutrition literacy is a behavioral skill that refers to the objective skills to effectively implement nutrition health behaviors, including the use of available health resources to access and understand nutrition information. Previous similar studies have pointed out that behavior change in an individual requires firstly knowledge about the disease, secondly awareness or attitude to change behavior, and finally the provision of certain behavioral skills, and when these three elements are combined, the goal of behavior change and health promotion is achieved (27).

## 6 Limitations

There are some limitations to this study: First, type 2 diabetes with verbal communication disorders and severe diseases were excluded, hence the scale was not validated in these populations. Second, this study used convenience sampling method and the sample was limited to Hangzhou city, China, and study population is relatively young and educated, which made the sample less representative and may affect the generalization and application of the scale. Besides, due to time constraints, we did not use the developed scale to assess the influencing factors of nutrition literacy in type 2 diabetes patients.

## 7 Recommendations for further research

In the future, firstly, researchers should conduct multicentre and large sample studies to determine the threshold values of the scale (e.g., what is a low score/what is a high score), which would be more meaningful for dissemination and clinical application of the scale. Secondly, the mean age of the patients in this study was 37.79 ± 13.65 and education literacy is mostly high school and above. Subsequent applications in low-income, low-education populations should validate the reliability of the tool and make necessary modifications for items that are difficult for patients to understand. Thirdly, the content of the scale items can be further strengthened. Many chinese believe in chinese medicine and use it extensively in their daily lives, and it is also used in the treatment of many diseases (32), so the scale can be improved by including knowledge related to nutrition in the items. In addition, longitudinal studies could assess how changes in nutrition literacy, as measured by the scale, impact diabetes management and health outcomes over time.

## 8 Implications for practice

This study provides clinical staff with a concise and reliable questionnaire to assess level of nutrition literacy in patients with type 2 diabetes. The type 2 diabetes nutrition literacy scale can be used to identify who are more prone to nutrition problems to adjust intervention programmes and additional monitoring. For example, for patients with low scores on the critical nutrition literacy dimension, health care providers can increase education on these topics. In addition, researchers can make cultural adaptations for use in different countries to help improve patient nutrition literacy levels. For example, this scale should be adapted to different languages through a standardized process, and the functional nutrient literacy dimensions should be matched and modified in conjunction with guidelines from different countries.

## 9 Conclusion

The nutrition literacy scale for patients with type 2 diabetes compiled in this study strictly followed the scale development process, resulting in 31 items in 4 dimensions with good reliability and validity, which can be used as a measurement tool to assess the nutrition literacy of patients with type 2 diabetes.

## Data Availability

The raw data supporting the conclusions of this article will be made available by the authors, without undue reservation.
